# Gli2 Acetylation at Lysine 757 Regulates Hedgehog-Dependent Transcriptional Output by Preventing Its Promoter Occupancy

**DOI:** 10.1371/journal.pone.0065718

**Published:** 2013-06-06

**Authors:** Sonia Coni, Laura Antonucci, Davide D'Amico, Laura Di Magno, Paola Infante, Enrico De Smaele, Giuseppe Giannini, Lucia Di Marcotullio, Isabella Screpanti, Alberto Gulino, Gianluca Canettieri

**Affiliations:** 1 CNRS UMR 7277, Inserm 1091, Institut de Biologie Valrose (iBV), Centre de Biochimie, Nice, France; 2 Université de Nice-Sophia Antipolis, Nice, France; 3 Department of Molecular Medicine, University of Rome “La Sapienza”, Rome, Italy; 4 Center for Life Nano, Istituto Italiano di Tecnologia, Rome, Italy Science@Sapienza; 5 Department of Experimental Medicine, University of Rome “La Sapienza”, Rome, Italy; 6 IRCCS Neuromed, Pozzilli, Isernia, Italy; University of Navarra, Spain

## Abstract

The morphogenic Hedgehog (Hh) signaling regulates postnatal cerebellar development and its aberrant activation leads to medulloblastoma. The transcription factors Gli1 and Gli2 are the activators of Hh pathway and their function is finely controlled by different covalent modifications, such as phosphorylation and ubiquitination. We show here that Gli2 is endogenously acetylated and that this modification represents a key regulatory step for Hedgehog signaling. The histone acetyltransferase (HAT) coactivator p300, but not other HATs, acetylates Gli2 at the conserved lysine K757 thus inhibiting Hh target gene expression. By generating a specific anti acetyl-Gli2(Lys757) antisera we demonstrated that Gli2 acetylation is readily detectable at endogenous levels and is attenuated by Hh agonists. Moreover, Gli2 K757R mutant activity is higher than wild type Gli2 and is no longer enhanced by Hh agonists, indicating that acetylation represents an additional level of control for signal dependent activation. Consistently, in sections of developing mouse cerebella Gli2 acetylation correlates with the activation status of Hedgehog signaling. Mechanistically, acetylation at K757 prevents Gli2 entry into chromatin. Together, these data illustrate a novel mechanism of regulation of the Hh signaling whereby, in concert with Gli1, Gli2 acetylation functions as a key transcriptional checkpoint in the control of morphogen-dependent processes.

## Introduction

The Hedgehog (Hh) pathway regulates development and stem/progenitor cell fate and its deregulation is a major cause of some malignancies, such as medulloblastoma, the most frequent pediatric solid tumor [1].

In mammals, Hedgehog signaling is activated by the interaction of the ligand with the inhibitory receptor Patched (Ptch). This alleviates the repression upon the transmembrane transducer Smoothened (Smo), which promptly migrates to the tip of the primary cilium, a microtubule-based organelle indispensable for Hedgehog function [2]. Once at the cilium, Smo triggers a cascade of events which leads to the activation of the three Gli transcription factors: Gli1, Gli2 and Gli3 [2]. Functionally, Gli1 and Gli2 are both activators, whereas Gli3 functions mainly as repressor of Hedgehog-dependent transcription.

Genetic studies have revealed that Gli2 and Gli3 are the primary mediators of Hh signaling and are essential for embryogenesis. Conversely, Gli1 is dispensable for development but plays a key role in tumorigenesis [3]. Indeed Gli1 and Gli2 possess transforming activity [4]–[6] and their levels are found elevated in Hedgehog-dependent tumors and other malignancies [7].

A key mechanism regulating Gli transcriptional activity is represented by post-translational modifications [3]. All three Glis are subjected to sequential phosphorylation and ubiquitination, but the consequences of these modifications differ among the three transcription factors. Gli3 and Gli2 are sequentially phosphorylated by protein kinase A (PKA), glycogen synthase kinase 3β (GSK3β) and casein kinase 1 (CK1). Once phosphorylated the two transcription factors are recruited by the F-box subunit of an SCF E3 ubiquitin ligase, βTrCP, which targets Gli2 and Gli3 to the proteasome, thus generating truncated N-terminal isoforms provided of repressive activity (GliR) over full length, active Gli (GliA). Activation of the Hh signaling prevents this partial proteolysis and favors the formation of the GliA isoform. The balance between GliA and GliR is finely regulated by the extracellular concentration of Hh ligands and represents a crucial mechanism to modulate the strength of Hh response.

Regulation of Gli2 and Gli3 processing is also regulated by interaction with Sufu that protects them from cullin/SPOP-mediated degradation [8]. Conversely, Gli1 cannot be cleaved into a repressor form and is not degraded by cullin3/SPOP, but is ubiquitinated and degraded by different ubiquitin ligases, in response to different conditions [9], [10].

Therefore, despite the three Glis share the regulation by phosphorylation and ubiquitination, these covalent modifications appear to be modulated by distinct kinases and ligases and to bring about different outcomes.

In our recent work, we have found that Gli1 is acetylated at a single conserved lysine, and that this modification inhibits its transcriptional activity [5]. Conversely, removal of acetylation by class I HDACs enhances Gli1-dependent gene expression, a process turned on by Hedgehog activation and limited by REN^KCTD11^ [5], [11] and other members of the KCASH family [12].

The mechanisms underlying the acetylation-dependent inhibition and the physiological conditions where this modification occurs are still not understood.

Given the relevance of Gli2 in mediating the transcriptional output of Hedgehog activation, here we have sought to understand the function and regulation of Gli2 acetylation.

We show that Gli2 is endogenously acetylated at a single conserved lysine and that this modification inhibits the transcriptional activity by preventing its promoter recruitment. Importantly, we provide evidence that Gli2 acetylation is a key conserved step, which regulates signal-dependent transcriptional activation and can be monitored during Hedgehog-mediated tissue development. Thus, in contrast to phosphorylation and ubiquitination, the acetylation/deacetylation checkpoint seems to operate though a conserved mechanism, involving an interplay between early and late activatory events.

## Materials and Methods

### Cell cultures and treatments

HEK293T and NIH3T3 cells were cultured as previously described [5]. For SAG treatment, NIH3T3 cells were incubated in low serum (0.5% bovine serum, BS) overnight, to allow a full Hedgehog response and then exposed to 200 nM SAG (Enzo Life Sciences) for 24 hours.

### Plasmid and site-directed mutagenesis

The following plasmids were provided by other laboratories: 8xGli-Luc reporter vector was provided by H. Sasaki, RIKEN Center for Developmental Biology, Japan; 12xGli-Luc reporter was from R. Tofgard, Karolinska Institutet, Sweden; Myc-Gli2 was from A. Dlugosz, University of Michigan, Ann Arbor, MI, U.S.A.; GST HAT-pCAF and PCI-p300 were provided by M. Fanciulli, Regina Elena Cancer Institute, Italy; TIP60 was provided by O. Segatto, Regina Elena Cancer Institute, Italy.

Myc-Gli2 K757R and K757Q were obtained through site directed mutagenesis (Quikchange II XL Site-directed mutagenesis kit, Stratagene) of the wild type Myc-Gli2 vector using the following primers:

hGLI2K757R forward: ACGCGGAACACCCAGCTGCCTC

hGLI2K757R reverse: GAGGCAGCTGGGTGTTCCGCGT

hGli2K757Q forward: ACGCGGAACACCAGGCTGCCTCCCCTC

hGli2K757Q reverse: GAGGGGAGGCAGCCTGGTGTTCCGCGT

Mutant sequences were verified by sequencing reactions.

### Transfection and Luciferase assay

HEK293T and NIH3T3 cells were transfected by using Lipofectamine 2000 Reagent or Lipofectamine Reagent and Plus reagent (Invitrogen) respectively, according to the manufacturer's protocol. Luciferase assays were performed as described previously [5], [13].

### Protein extractions, Nucleus/Cytoplasm extraction and Immunoblots

Cells were lysed with RIPA buffer (0.5% sodiumdehoxycolate, 50 mM Tris HCl pH 7.6, 1% NP40, 0.1% SDS, 140 mM NaCl, 5 mM EDTA pH 8, 5 mM sodium pyrophosphate, 5 mM sodium butyrate) supplemented with protease inhibitors and total protein extracts were quantified. For nucleus and cytoplasm extraction, cells were lysed in 100 µL Buffer A (10 mM HEPES pH 7.4, 10 mM KCL, 10 mM NaCl, 0.1 mM EDTA, 0.1 mM EGTA, 1 mM DTT, 0.5 mM PMSF) and incubated on ice. 0.6% NP40 was added to the samples. After centrifugation, the supernatant (cytoplasmic fraction) was removed and the pellet resuspended into 500 µL Buffer B (20 mM HEPES pH 7.4, Glycerol 20%, KCl 100 mM, EDTA 1 mM, DTT 1 mM, PMSF 0.5 mM, Leupeptin 10 ug/mL) and incubated on ice. 1% NP40 was added and samples centrifuged. The pellet was resuspended into 50 µL Buffer C (20 mM HEPES pH 7.4, Glycerol 20%, 400 mM NaCl, EDTA 1 mM, EGTA 1 mM, PMSF 0.5 mM, DTT 1 mM, Leupeptin 10 µg/µL), incubated on ice and centrifuged to isolate the nuclear fraction.

Protein extracts were analyzed by SDS-PAGE and blotted onto a nitrocellulose membrane (Perkin Elmer). Membranes were blocked by 5% milk in Tris buffered saline with 0.1% Tween20, and incubated with primary antibodies.

The following antibodies were used: rabbit polyclonal antibody against acetylated lysine (1∶1000, Upstate, 06933), goat anti Gli2 (1∶1000, R&D systems), anti-Gli2 (C-10) (Santa Cruz, sc-271786), mouse anti Myc-HRP (1∶ 2000, Sigma), mouse anti-Myc (1∶1000, Sigma, M4439), anti Acetyl-Gli2(Lys757) (1∶1000) anti-Tubulin (1∶1000, SantaCruz sc-8035), anti-CREB (1∶2000, gift from M. Montminy, Salk Institute La Jolla, CA).

Signals were detected using Western Lightning *Plus*-ECL (Perkin Elmer).

### Generation of Acetyl Gli1 and Gli2 antisera

Acetyl-Gli2(Lys757) and Acetyl-Gli1(Lys518) antisera were generated by Eurogentec by rabbit immunization with specific peptide:

Acetilated-Gli1(Lys518) H2N-IGS RGL K(Ac)LPSLT C- CONH2

Acetilated-Gli2(Lys757) H2N-PHTRNTK(Ac)LPPLPC-CONH2

The specificity of the antibodies was validated by competition assays with the immunogenic peptides, with or without lysine acetylation.

### Acetylation assay

Acetylation assays were performed as previously described [5].

HEK293T, transfected with the indicated plasmids, were lysed and overexpressed Gli2 was immunoprecipitaded with anti Myc (9E10) AC beads (Santa Cruz, sc-40 AC); NIH3T3 cells were lysed and endogenous Gli2 was immunoprecipitated with goat anti-Gli2 (1∶100, R&D systems). After immunoblott acetylation was detected with rabbit polyclonal anti acetyl lysine (1∶1000, Upstate, 06933), or anti Acetyl-Gli2(Lys757) (1∶1000).

### RNA analysis and quantitative PCR

Total RNA was extracted from NIH3T3 cells, reverse transcribed and analized by quantitative Real Time PCR as previously described [5], using the following ABI TaqMan probe: mouse *Ptch1* (Mm00436026_m1), *GAPDH* (Mm99999915_G1), *HPRT* (Mm00446968_m1), *GLI1* (Mm00494645_m1).

### Chromatin immunoprecipitation (ChIP)

HEK293T cells, transfected with the indicated coding vectors, were crosslinked and chromatin immunoprecipitation was carried out as previously described [5] with mouse anti-Myc antibody (1∶100, Sigma, M4439). Eluted DNA was analyzed with quantitative Real Time PCR as described [14] using the following primers:

hPtch1 promoter, forward: 5-GTATTGCATGCGAGAAGGTTGG-3;

hPtch1 promoter, reverse: 5-TTTCTGCGACGCGATTGGCTCG-3;

hGAPDH promoter, forward: 5-AGAACATCATCCCTGCCTCT -3;

hGAPDH promoter, reverse: 5-CACCTGGTGCTCAGTGTAG -3.

Data are shown as fold difference vs Empty vector.

### Immunohistochemistry

Immunohistochemistry was performed as described previously [5].

Sections from paraffin-embedded tissues were deparaffinized with xylol, fixed with decreasing ethanol concentrations, blocked and incubated with anti Gli1 (H-300, 1∶200, SantaCruz Biotechnology, SC-20687), or anti acetyl-Gli1(Lys518) or anti acetyl-Gli2(Lys757) antibodies (1∶50). Sections were counterstained with hematoxylin.

### Statistical analysis

Statistical analysis was performed using StatView 4.1 software (Abacus Concepts, Berkeley, CA). Results are expressed as mean ± s.d. of at least three separate experiments, each performed in triplicate. Statistical differences were analyzed with the Mann-Whitney *U* test for non-parametric values and a p<0.05 was considered significant.

## Results

### p300 inhibits Gli2 activity by acetylating lysine 757

**Figure 1:**
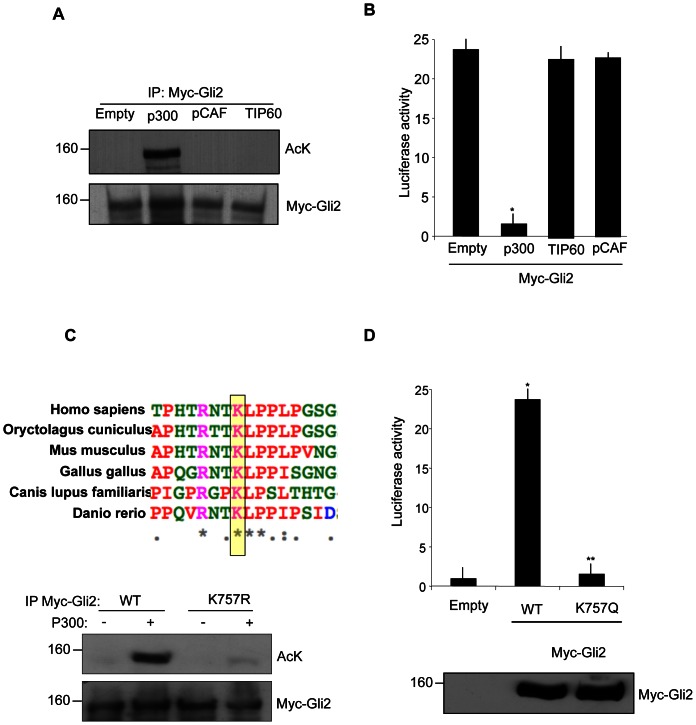
p300 acetylates Gli2 at the conserved Lysine 757. (**A**) In vivo acetylation assay in HEK293T cells transfected with plasmids coding for Myc tagged Gli2 and the indicated HATs. Cell extracts were immunoprecipitated with anti-Myc antibody and acetylation was revealed with anti-acetyl lysine antisera. Western blot analysis with anti Myc antibody showed equivalent amounts of Myc-Gli2. Empty, pcDNA3. (**B**) Luciferase assay in HEK293T. Cells were transfected with 12× Gli-Luc and TK renilla reporter vectors, Gli2, p300, TIP60, pCAF where indicated. *p<0.01 vs Empty (pcDNA3). Results are shown as the average ± SD of triplicate experiments (n = 4). (**C**) (Top) ClustalW alignment of the region surrounding lysine 757 of Gli2 in different species. Identical, strongly conservative and weakly conservative aminoacids are indicated by asterisk, colon and dots respectively, according to ClustalW convention. (Bottom) In vivo acetylation assay in HEK293T cells transfected with Myc-Gli2 WT or K757R and p300 where indicated. Acetylation was detected as previously described. (**D**) (Top) Luciferase assay performed in HEK293T cells transfected with 12× Gli-Luc, TK renilla, Myc-Gli2 WT or the acetylation mimetic mutant Myc-Gli2 K757Q. *p<0.01 vs Empty; **p<0.05 vs WT. Results are shown as the average ± SD of triplicate experiments (n = 3). (Bottom) Western blot analysis of Myc-Gli2 WT and K757Q expression levels.

We first tested the ability of different histone acetyltransferases (HATs) to acetylate Gli2. We selected three different HATs, each representing a member of the main HAT families [15]: p300 (p300/CBP family), TIP60 (MYST family) and pCAF (GNAT family). Overexpression of p300 induced a robust acetylation of the exogenous Gli2 (Fig. 1A), whereas Tip60 and pCAF did not change the acetylation status of Gli2, indicating the specificity of p300 acetyltransferase in this process.

To study the functional consequence of Gli2 acetylation, we performed luciferase assays with a Gli responsive reporter (12× Gli-Luc) and the different HATs: exogenous p300 caused a strong inhibition of Gli2 mediated reporter activity, while TIP60 and pCAF did not display any effect (Fig. 1B). Thus, p300 induces acetylation of Gli2, accompanied by a decrease of its transcriptional activity.

Gli2 contains a potentially acetylatable lysine (K757), which is conserved in Gli1 (K518) but not in Gli3 [5]. This lysine is also evolutionary conserved among phylogenetically distant species (Fig. 1C, top).

To determine whether lysine 757 is acetylated, we mutated it into arginine and performed in vivo acetylation assay with the mutant Gli2K757R. As shown in Figure 1C, mutation of K757 abrogated the ability of Gli2 to be acetylated by p300, demonstrating that this is the only acetylatable residue of Gli2.

It has been shown that substitution of a lysine with a glutamine mimics a status of constitutive acetylation [16]. Thus, to verify the effect of acetylation at K757 we generated a K757Q mutant. Supporting the hypothesis that acetylation of Gli2 is an inhibitory modification, K757Q substitution prevented almost completely the Gli-dependent transcriptional activation, despite the levels of the expressed proteins were identical (Fig. 1D).

### Endogenous Gli2 is deacetylated in response to Hedgehog activation

Having observed that Gli2 acetylation at K757 inhibits its transcriptional activity, we next wondered whether this modification occurs at endogenous level and how it is connected with the activation status of the Hedgehog pathway.

**Figure 2:**
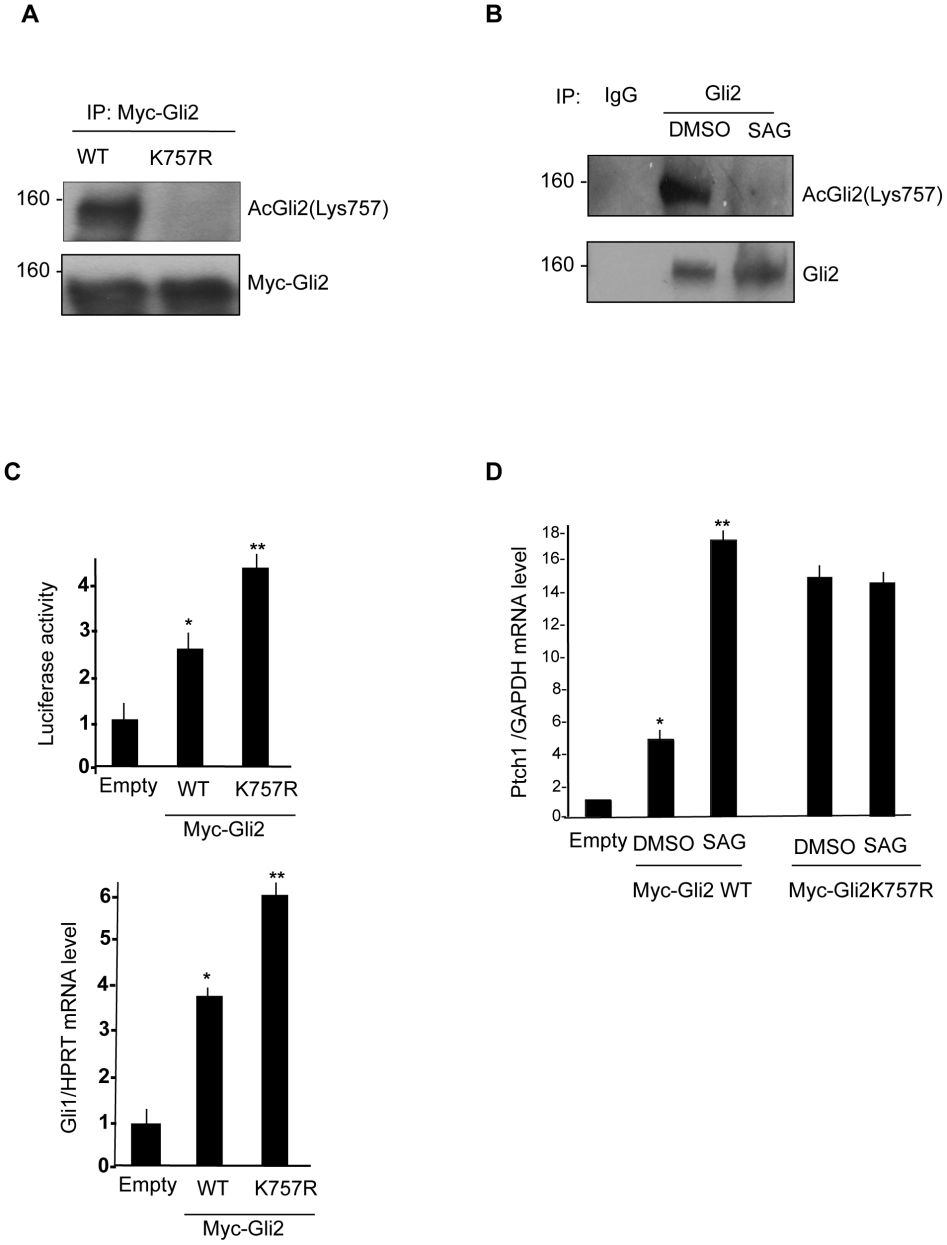
Removal of Gli2 acetylation enhances Hh-dependent transcription. (**A**) K757 acetylation of wild type Myc tagged Gli2 and K757R mutant in HEK293T cells. Cells extract were immunoprecipitated with anti-Myc antibody and acetylation was detected by anti acetyl-Gli2(Lys757) specific antisera. Western blot analysis with anti Myc antibody showed equivalent amounts of Myc-Gli2. (**B**) Acetylation of endogenous Gli2 in NIH3T3 cells, treated with SAG or DMSO for 24 hours. Acetylation was detected with anti acetyl-Gli2(Lys757) specific antisera. (**C**) (Top) Luciferase assay in NIH3T3 cells transfected with 8× Gli-Luc reporter, Myc tagged Gli2 wild type and K757R mutant. *p<0.01 vs Empty. Results are shown as the average ± SD of triplicate experiments (n = 3). (Bottom) *Gli1* mRNA levels normalized with the housekeeping *HPRT* mRNA in NIH3T3 cells transfected with Myc-Gli2 WT and K757R mutant. (**D**) Transcriptional activity of Myc-Gli2 WT and K757R mutant in response to SAG treatment: *Ptch1* mRNA levels (QPCR), normalized with the housekeeping *GAPDH* mRNA in 24 hours SAG-treated NIH3T3 cells, transfected with Empty vector, Myc tagged Gli2 wild type and K757R mutant. *p<0.01 vs Empty, **p<0.05 vs DMSO; Results are shown as the average ± SD of triplicate experiments (n = 3).

Toward this end, we generated a rabbit polyclonal antibody against the acetylated K757 of Gli2, which was able to readily detect this modification and did not react against K757R mutant Gli2 (Fig. 2A).

To determine whether acetylated Gli2 is detectable at endogenous levels and to study if this modification is perturbed in response to activated Hedgehog signaling, we performed acetylation assays in NIH3T3 cells treated with the Smo agonist SAG. As shown in Figure 2B, following immunoprecipitation, endogenous acetylated Gli2 was promptly detected with anti acetyl-Gli2(Lys757) and exposure of cells to the Smo agonist SAG caused a significant decrease of K757 acetylation.

To study the contribution of K757 deacetylation to the Gli2-dependent transcriptional activation, we analyzed the activity of K757R Gli2 mutant, mimicking a status of constitutive deacetylation in luciferase assays and by quantitative Real Time PCR (Fig. 2C). Compared to the wild type protein, ectopic expression of K757R mutant induced a stronger luciferase activity (Fig. 2C, top) and upregulation of endogenous Gli1 mRNA levels (Fig. 2C, bottom), indicating a gain of function activity. Together, these data demonstrate that removal of Gli2 acetylation enhances Hh-dependent transcription.

We next performed a quantitative Real Time PCR to assess the levels of the Hh target mRNA *Ptch1*, in NIH3T3 cells transfected with Gli2 WT or K757R mutant, and treated with SAG or vehicle control.

Exogenous expression of Gli2 caused a significant five fold increase of *Ptch1* transcript that was further increased by four fold upon Smo activation with SAG (Fig. 2D). In keeping with the luciferase data, expression of K757R mutant showed a significantly higher transcriptional activity compared to WT Gli2. Conversely, SAG failed to further increase the activity of K757R mutant construct, indicating that abrogation of K757 acetylation prevents the ability of SAG to modulate Gli2 transcriptional activity.

We next studied if the acetylation/deacetylation balance plays a role in biological contexts regulated by the Hh signaling. We selected the developing cerebellum of 6 days old mice (P6), where Hh pathway regulates development of the cerebellar granule cell progenitors and promotes their mitotic expansion in the External Granular Layer (EGL).

**Figure 3:**
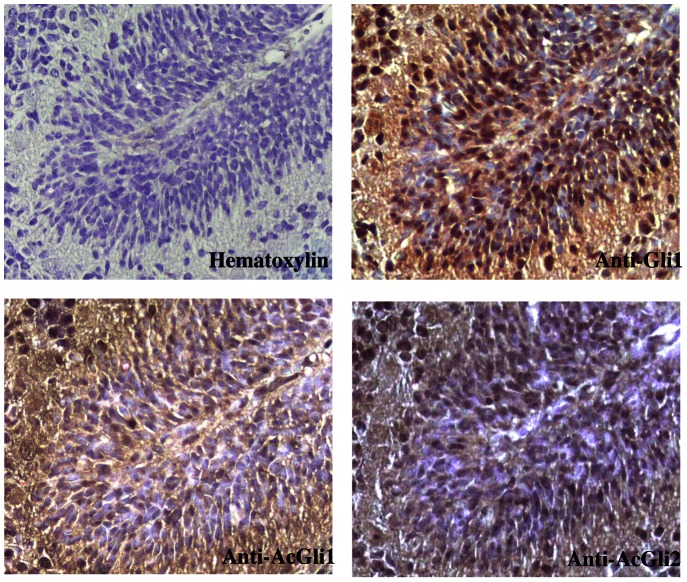
Acetylation of Gli transcription factors is not detectable in the External Granular Layer. Immunohistochemical staining of Gli1(upper-right), acetyl-Gli1(Lys518) (bottom-left), acetyl-GLi2(Lys757) (bottom-right), or Hematoxylin (upper-left) in a 6-day-old mouse cerebellum.

Confirming the increased Hh activation status, incubation of sections with anti Gli1 antisera, strongly stained the EGL (Fig. 3). In contrast, neither acetylated Gli1 nor acetylated Gli2 were detectable in the same region, thus indicating that both these Hh transcriptional activators are deacetylated in the EGL at this stage of development.

### Acetylation of Gli2 prevents its promoter occupancy

Activation of Gli2 function involves at least three main steps: i) a modification of processing/stability with alterations of its half–life; ii) changes in cellular compartmentalization; iii) interaction with target promoters and transcriptional cofactors to induce transcription. Therefore, we next addressed whether acetylation of Gli2 affects one of these events leading to transcriptional activation.

**Figure 4:**
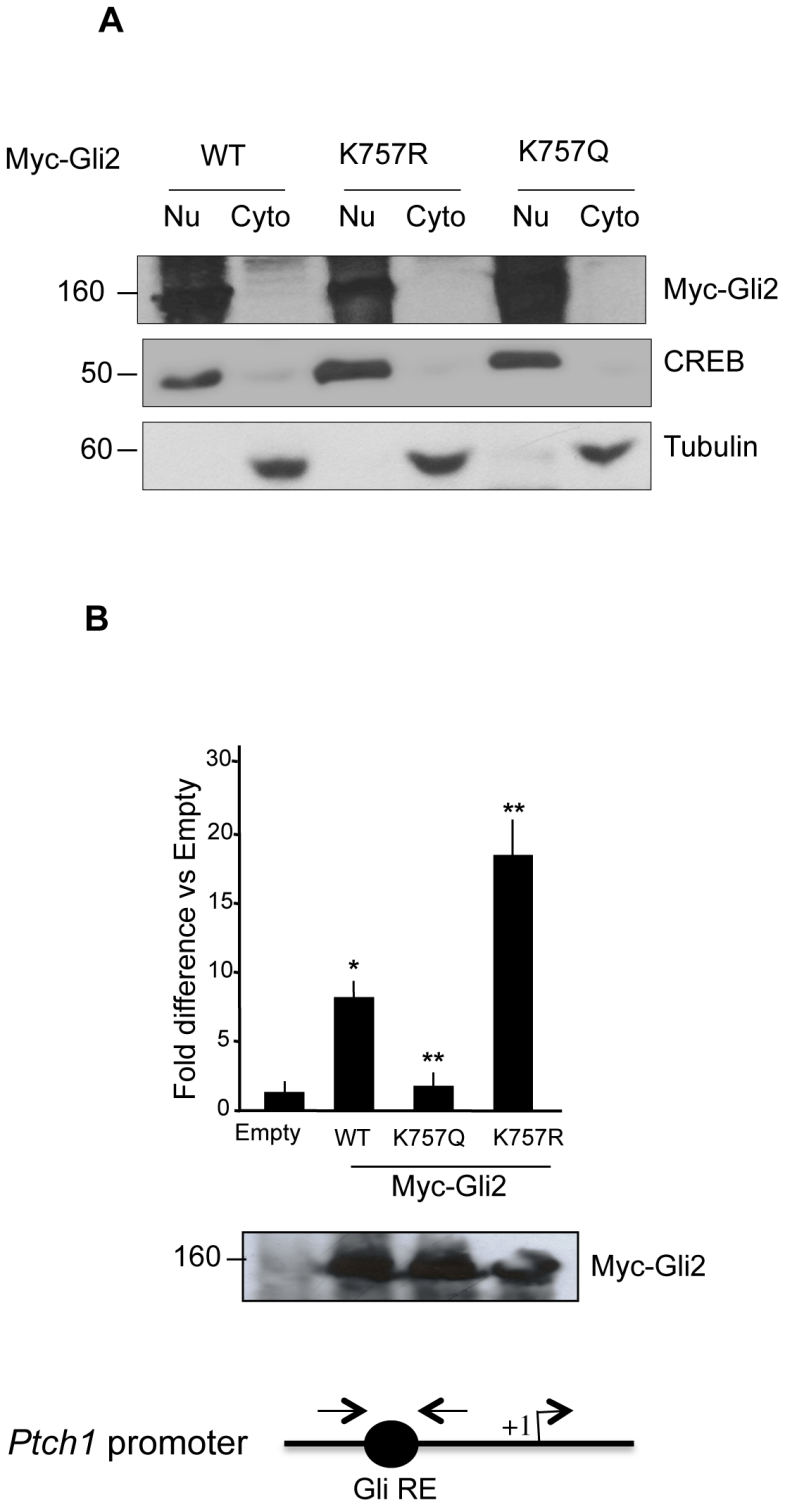
Gli2 acetylation prevents its promoter occupancy. (**A**) Cellular fractionation of NIH3T3 cells shows no difference in the localization of Myc tagged Gli2 wild type (WT), K575R and K575Q mutants. Purity of fractionation documented by tubulin (cytoplasmic, Cyto) and CREB (nuclear, Nu) staining. (**B**) Promoter occupancy of Gli2 is prevented by K757 acetylation. HEK293T cells were transfected with Myc tagged Gli2 wild type (WT), K757R, K757Q and Empty vectors and Chromatin Immunoprecipitation (ChIP) was carried out. Quantitative real-time PCR was performed using primers encompassing the Gli-BS of human *Ptch1* promoter (bottom, schematic representation). Results are indicated as fold difference, relative to Empty (pcDNA3) control. *p<0.05 WT vs Empty, **p<0.05 K757Q vs WT. Results are shown as the average ± SD of triplicate experiments (n = 4). Western blot analysis with anti Myc antibody showing equivalent amounts of Myc-Gli2 WT and mutants. Empty, pcDNA3.

We ruled out the possibility that acetylation could interfere with Gli2 stability since the steady state levels of WT and mutant proteins did not appear to be different (Fig. 4B bottom). Thus, we studied if the inhibitory effect of acetylation could be attributed to a change in intracellular localization. To this end, we analyzed the nuclear and cytoplasmic localization of WT and mutant Gli2 plasmids. As shown in Figure 4A, Gli2 WT, K757R and K757Q mutants did not show differences in their cellular localization, which appeared to be predominantly nuclear. This data suggested that inhibition of Gli2 by acetylation is a nuclear process, which is likely to interfere with one of the transactivating steps occurring at the chromatin level. To address this issue we performed chromatin immunoprecipitation assays in cells transfected with Gli2 WT and mutant plasmids and analyzed their recruitment over the Gli-responsive element of the *Ptch1* promoter [5].

Remarkably, the promoter occupancy of Gli2 K757Q was abrogated, compared to WT Gli2, whereas the K757R mutant displayed a significant increase in chromatin binding (Fig. 4B). Therefore, these data suggested that acetylation inhibits Gli2 activity by preventing its chromatin recruitment.

## Discussion

Protein acetylation is a critical regulatory step for histones and many non-histone proteins, including transcription factors, enzymes or structural proteins [17].

In the present work we have characterized the acetylation of Gli2, the early transcriptional effector of Hedgehog signaling. We have demonstrated that Gli2 is acetylated at lysine 757 by the co-activator p300 and that this modification inhibits Hh-target gene expression.

While it is still unclear the type of signals that may induce this p300-mediated acetylation of Gli2, we have shown that removal of the K757 acetyl group occurs upon activation of Hh signaling. It is likely that the mechanism linking Hh activation to Gli2 deacetylation involves the activation of class I HDACs [5], which are induced by Smo agonists at transcriptional and, perhaps, post-transcriptional level.

Interestingly, the acetylation/deacetylation equilibrium seems to contribute to the typical dynamic response of Hh-dependent transcription. Indeed, a Gli2 mutant mimicking a constitutive deacetylation status is hyperactive but no longer induced by Smo agonists.

Thus, in addition to the balance between the full length GliA and the truncated GliR isoforms, the acetylation/deacetylation balance seems to be an additional regulated mechanism to switch from one status to another. This observation adds further complexity to the classical model of Gli activation and implies that acetylation represents a supplementary mechanism utilized by cells to modulate Hedgehog output. In support to this model, we show that, when monitored with a specific antibody, acetylated Gli2 cannot be found in territories of active Hedgehog signaling, such as the outer EGL, where HDAC1 levels have also been found to be upregulated [5].

It will be important to understand whether and to what extent acetylation also contributes to the graded response to ligand concentrations, thus representing a further level of control during morphogen dependent development.

What are the mechanisms of transcriptional inhibition following Gli2 acetylation? In this work we have demonstrated that acetylation of Gli2 does not affect its intracellular localization and stability. Instead, acetylation appears to prevent Gli2 recruitment to target chromatin. Since the residue K757 maps outside the zinc finger domain, it is unlikely that acetylation affects Gli2/DNA affinity. A plausible explanation is that K757 acetylation prevents the association with chromatin-bound cofactors, which are essential for the promoter entry/transactivation of Gli2. In this regard, a potential candidate for this effect could be the ATP-dependent chromatin remodeling factor Brg, which has been reported to interact with both HDACs and Glis and to participate to their Hh-induced chromatin recruitment and target gene activation [18]. Further work is needed to demonstrate these issues.

**Figure 5:**
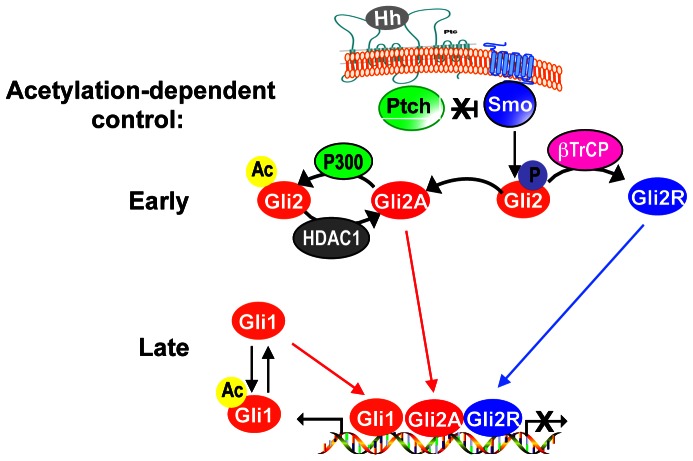
Model of acetylation-dependent control of Gli activity. Following Hh/Ptch interaction, Smo triggers a signaling cascade leading to Gli2 deacetylation and to the inhibition of the βTrCP-regulated balance between Gli2R and full length Gli2 (Gli2A). Both events contributes to the early signal-dependent activation of the Hh pathway. Once activated, Gli2 promotes transcription of Gli1, whose activity is also regulated by Hh-induced HDAC1-mediated deacetylation, thus generating a positive feedback loop (late activation).

In conclusion, these data illustrate a novel mechanism of regulation of the Hh signaling, where acetylation of Gli2 at lysine 757 functions as a critical regulated step, controlling the activation status of Hh pathway. We suggest that this mechanism contributes to the early regulatory events immediately downstream of Smo (i.e. cleavage-dependent balance between Gli2A and Gli2R), and is functionally coordinated with Gli1 acetylation, which represents a late regulated step of Hedgehog pathway activation (Fig. 5).

These results further emphasize the relevance of Gli acetylation as a key regulatory epigenetic modification, with promising therapeutic potential for diseases linked to aberrant Hh pathway activation.
